# A Case of Ketorolac-Induced Aseptic Meningitis

**DOI:** 10.7759/cureus.57349

**Published:** 2024-03-31

**Authors:** Lauren Shahin, Azjaah Rogers, Leroy Swain, Felicia F Ourn, Tye Barber

**Affiliations:** 1 Family Medicine, Broward Health Medical Center, Fort Lauderdale, USA; 2 Family Medicine, Nova Southeastern University Dr. Kiran C. Patel College of Osteopathic Medicine, Fort Lauderdale, USA; 3 Internal Medicine, Broward Health Medical Center, Fort Lauderdale, USA

**Keywords:** nonsteroidal anti-inflammatory drugs (nsaids), meningeal sign, cerebrospinal fluid (csf), aseptic meningitis, drug-induced aseptic meningitis

## Abstract

Drug-induced aseptic meningitis is a rare condition that occurs because of an adverse reaction to medications such as nonsteroidal anti-inflammatory drugs (NSAIDs) and antibiotics. Unlike bacterial or viral meningitis, aseptic meningitis is not caused by an infection, but rather by an inflammatory response. This condition creates a challenge since patients with aseptic meningitis often present with classic clinical meningeal symptoms, including fever, headache, and neck stiffness. We present a case of a patient with NSAID-induced aseptic meningitis and highlight the importance for healthcare providers to have a high index of suspicion for drug-induced aseptic meningitis in patients presenting with symptoms of meningitis with negative cerebrospinal fluid analysis and culture.

## Introduction

Aseptic meningitis refers to the inflammation of the meninges in patients who have negative cerebrospinal fluid (CSF) bacterial cultures. Aseptic meningitis can stem from both infectious and non-infectious causes [1]. The infectious etiologies include enteroviruses, fungi, and parasites. Enteroviruses are the most common pathogens in immunocompetent individuals [2]. Non-infectious aseptic meningitis is usually caused by underlying pro-inflammatory medical conditions, including neoplasms, systemic lupus erythematosus, and sarcoidosis. Systemic lupus erythematosus stands as the most frequent underlying condition associated with drug-induced aseptic meningitis [3].

Drug-induced aseptic meningitis is a rare condition and a diagnosis of exclusion. The incidence of drug-induced aseptic meningitis is unknown and the pathogenesis is not fully understood [4,5]. The most common agents involved in drug-induced aseptic meningitis are nonsteroidal anti-inflammatory drugs (NSAIDs), antibiotics, intravenous immunoglobulins, and immunosuppressive drugs [3]. NSAIDs are one of the most used agents for their anti-inflammatory, antipyretic, and analgesic properties. However, one of the neurologic side effects of NSAIDs is aseptic meningitis [6]. Ibuprofen is the most common NSAID that causes aseptic meningitis, but other NSAIDs such as diclofenac and naproxen have been associated with aseptic meningitis [7]. We present a unique case of aseptic meningitis induced by ketorolac (Toradol), an NSAID used widely for pain management.

## Case presentation

**Figure 1:**
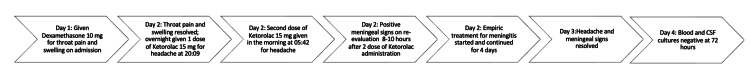
Timeline of main events

A 63-year-old male with diabetes and hypertension presented to the emergency room with swelling of the throat occurring the morning of the presentation. He was evaluated at a medical center prior to arrival where he was given diphenhydramine 50 mg IV once, famotidine 20 mg IV once, and methylprednisolone 125 mg IV once and discharged with suspected angioedema due to angiotensin-converting enzyme (ACE) inhibitor. A week earlier, he also had swelling in the throat that resolved after taking over-the-counter Allegra. The patient denied any known allergies to food, medications, or environmental factors. He denied having had any allergy testing done in the past. He admitted to compliance with lisinopril 20 mg every day for the past three years with no issues. There was no significant family history reported. The timeline of the main events of the case is illustrated in Figure 1.

On examination, he was afebrile with a temperature of 36.6°C, blood pressure of 186/91 mmHg, pulse of 98 bpm, respiratory rate of 20 breaths per minute, and oxygen saturation of 97% on room air. His physical exam was remarkable for left submental lymphadenopathy that was tender to palpation and an erythematous soft palate. Laboratory tests revealed a total white blood cell count of 6,170/mL with a differential of 88.7% neutrophils and 10% lymphocytes. The absolute eosinophil count was in the normal low range. COVID-19, rapid strep, and mononucleosis tests were negative and procalcitonin level was unremarkable. A noncontrast computed tomography (CT) of the neck with contrast showed that the larynx was slightly asymmetric with some soft tissue swelling of the left vocal cords. The patient was given dexamethasone 10 mg and hydralazine 20 mg in the emergency room and admitted for clinical surveillance. His home blood pressure medication, lisinopril, was held to rule out drug-induced angioedema.

On the first day of admission, the otolaryngology consult evaluated the patient and cleared him for discharge after the throat pain and swelling resolved. However, earlier that morning, the patient had a low-grade fever of 37.9°C and he complained of a generalized headache, for which he was subsequently given ketorolac 15 mg IV twice. The patient received a total of 30 mg of ketorolac within 48 hours. Upon re-evaluation, the patient was found to have nuchal rigidity with positive Kernig’s sign, Brudzinski’s sign, and Jolt accentuation. Meningeal signs were present approximately eight to 10 hours after the second dose of ketorolac was administered. The rest of his physical exam was otherwise unremarkable. Because the patient had concerning symptoms of meningitis, empiric treatment was initiated with antibiotic and antiviral therapy, and a prompt lumbar puncture order was placed for a definitive diagnosis. Blood cultures were drawn prior to initiation of treatment with vancomycin, ceftriaxone, ampicillin, and acyclovir. A loading dose of vancomycin 1750 mg was administered, followed by vancomycin 1250 mg IV bid for one day and vancomycin 1500 mg IV twice daily for two days, ceftriaxone 2 gm IV twice daily for four days, ampicillin 2 gm IV six times daily for four days, and acyclovir 700 mg IV three times daily for four days. Isolation precautions were followed per the Centers for Disease Control and Prevention (CDC) guidelines [8].

By the second day of admission, the throat pain and swelling had completely resolved, and the patient’s headache improved. No meningeal signs were present on the physical exam. A noncontrast CT scan of the head was done prior to the lumbar puncture to rule out any intracranial abnormality. Complete CSF analysis is presented in Table 1. The lumbar puncture was significant for white blood cell (WBC) count of 11 cells/mm^3^ (normal range: 0-5 cells/mm3) and CSF glucose of 97 mg/dL (normal range: 40-70 mg/dL). CSF protein, red blood cell (RBC), polymorphs, monocytes, and lymphocytes were within normal limits. Infectious disease was consulted and recommended discontinuing NSAIDs for suspected drug-induced aseptic meningitis. Ketorolac was promptly discontinued, and the patient improved without specific intervention. The patient received empiric meningitis treatment for four days until the blood and CSF cultures were negative after 72 hours and the CSF meningitis and encephalitis polymerase chain reaction (PCR) panel and cryptococcal tests came back negative. The patient was optimized for discharge with follow up instructions and the presumptive diagnosis of aseptic meningitis induced by NSAIDs. Preliminary screening for autoimmune disease with antinuclear antibody was negative.

**Table 1:** Cerebrospinal fluid analysis CSF, cerebrospinal fluid; WBC, white blood cells; RBC, red blood cells; Polys, polymorphonuclear cells; Lymph, lymphocytes; Mono, monocytes; CM, cytomegalovirus; EV, enterovirus; HSV, herpes simplex virus; VZ, varicella zoster virus; HHV6, human herpesvirus 6; HPeV, human parechovirus; PCR, polymerase chain reaction.

Lab test	Lab values	Reference values
CSF color	Colorless	Colorless
CSF appearance	Clear	Clear
CSF glucose level (mg/dL)	97 (H)	40-70
CSF protein (mg/dL)	30	15-40
CSF WBC (cells/mm^3^)	11 (H)	0-5
CSF RBC (cells/mm^3^)­­­­	5	0-5
Polys CSF (%)	92	NA
Lymph CSF (%)	0	NA
Mono CSF (%)	7	NA
Cryptococcal Ag	Negative	NA
Meningitis encephalitis panel PCR (CM, EV, HSV, VZ, HHV6, HPeV, *Streptococcus pneumonia*, *Streptococcus agalactiae*, *Cryptococcus gattii*, *Haemophilus influenzae*, *Listeria monocytogenes*)	Negative	NA
CSF culture	No growth after 72 hours	NA

## Discussion

Drug-induced aseptic meningitis should be suspected in a patient who presents with symptoms consistent with meningitis, including fever, headache, nuchal rigidity, and altered mental status, especially if an underlying cause is not identified and the symptoms began after drug exposure [5]. It is also important to keep in mind that a number of patients with meningitis may not present with meningeal signs on physical exam [9]. Therefore, if there is a clinical suspicion of bacterial meningitis, antibiotic therapy should be initiated until the cultures are negative.

The pathogenic mechanism of NSAID-induced aseptic meningitis does not appear to be mediated by the inhibition of prostaglandin synthesis [10]. There are two proposed theories for the mechanism by which NSAIDs can cause drug-induced aseptic meningitis: a hypersensitivity reaction and direct irritation of the meninges. Desgranges et al. state that the physiopathology mechanisms of drug-induced aseptic meningitis remain poorly understood because of the various eliciting drugs and the condition of the patient [11]. This is further highlighted in the different types of pleocytosis that have been reported in the CSF analysis [11]. The typical CSF findings found in aseptic meningitis are normal to mildly decreased glucose, normal to mildly elevated protein, and a cell count of 10-1000 cells per microliter that is initially neutrophil predominant with a gradual shift toward lymphocyte predominant [1]. However, a spectrum of CSF findings and clinical presentations has been recorded. CSF findings in ibuprofen-induced aseptic meningitis vary [2]. Our patient demonstrated a pleocytosis and elevated glucose level, as shown in Table 1.

In a review paper by Moris et al., NSAIDs, particularly ibuprofen, were associated with the highest number of cases of drug-induced aseptic meningitis [3]. While some studies have reported that aseptic meningitis is independent of the dose of NSAID, others have shown that doses of ibuprofen as low as 200 mg orally can elicit this adverse reaction [11,12]. The interval between drug intake and the development of meningitis was also shown to vary between several minutes to four months [3]. The prognosis for drug-induced aseptic meningitis is usually good [5]. Early diagnosis is important since the cessation of the antibiotic leads to rapid clinical improvement [4]. According to Pires et al., symptoms typically resolve rapidly within one to five days after drug withdrawal [13]. Drug-induced aseptic meningitis can occur in patients who have previously tolerated the offending drug [13].

## Conclusions

Our case report highlights the importance of excluding all other causes of meningitis and obtaining a thorough medication history before diagnosing a patient with drug-induced aseptic meningitis. As this condition is rare, it is crucial for healthcare providers to be aware of the side effects of NSAIDs. Clinicians should maintain a high index of suspicion for drug-induced aseptic meningitis as early diagnosis and withdrawal of the offending agent leads to a rapid reversal of symptoms. It is important that patients with drug-induced aseptic meningitis are screened for autoimmune diseases and made aware to avoid all NSAIDs to prevent reoccurrence.
